# All grown up? The fate after 15 years of a quarter of a million UK firms born in 1998

**DOI:** 10.1007/s00191-017-0549-x

**Published:** 2018-01-09

**Authors:** Michael Anyadike-Danes, Mark Hart

**Affiliations:** Aston Business School, Birmingham, UK

**Keywords:** Firm growth, Firm age, Firm size, Firm survival, Birth cohort, L25, L26, E24, M13

## Abstract

The theory of firm growth is in a rather unsatisfactory state. However, the analysis of large firm-level datasets which have become available in recent years allows us to begin building an evidence base which can, in turn, be used to underpin the development of more satisfactory theory. Here we study the 239 thousand UK private sector firms born in 1998 over their first 15 years of life. A first, and quite striking, finding is the extraordinary force of mortality. By age 15, 90% of the UK firms born in 1998 are dead, and, for those surviving to age 15, the hazard of death is still about 10% a year. The chance of death is related to the size and growth of firms in an interesting way. Whilst the hazard rate after 15 years is largely independent of size at birth, it is strongly affected by the current (age 14) size. In particular, firms with more than five employees are half as likely to die in the next year as firms with less than five employees. A second important finding is that most firms, even those which survive to age 15, do not grow very much. By age 15 more than half the 26,000 survivors still have less than five jobs. In other words, the growth paths – what we call the ‘growth trajectories’ – of most of the 26,000 survivors are pretty flat. However, of the firms that do grow, firms born smaller grow faster than those born larger. Another striking finding is that growth is heavily concentrated in the first five years. Whilst growth does continue, even up to age 15, each year after age five it involves only a relatively small proportion of firms. Finally, there are two groups of survivors which contribute importantly to job creation. Some are those born relatively large (with more than 20 jobs) although their growth rate is quite modest. More striking though, is a very small group of firms born very small with less than five jobs (about 5% of all survivors) which contribute a substantial proportion (more than one third) of the jobs added to the cohort total by age 15.

## Introduction

The theory of firm growth is in a rather unsatisfactory state. Indeed a reading of some recent contributions to the literature suggests that there may be something of an impasse. To summarise, albeit somewhat crudely, one view is that firm growth (certainly in a firm’s early years) can be regarded as essentially ‘random’; the alternative view is that a firm’s characteristics and its choices can have a systematic effect on its growth. Whilst these competing views provide a context for this paper, our starting point is different. We propose to make use of a relatively newly available large firm-level dataset to begin building an evidence base which, by providing a detailed picture of growth across the UK firm population, might be used to underpin the development of more satisfactory approach to the theory of firm growth.

One of the most striking findings from following a birth cohort of firms, as we do here, is the extraordinary force of mortality: by age 15, 90% of a cohort of UK firms born in 1998 are dead; and, of those that do survive to age 15, the hazard of death is still about 10% a year. It is against this background that we investigate the growth paths – what we call the ‘growth trajectories’ – over 15 years of the 26 thousand survivors of the 239 thousand firms born into the cohort. We find that very few of the 26 thousand 15 year survivors grow very much, but of those that do, smaller firms grow faster than larger. Moreover for most of the surviving firms that do grow, fast growth is concentrated in the first five years.

The key data analytical device which we use to track survival and growth, is a classification of firms into size-bands. We then follow firms progress (or demise) using a series of ‘Origin/Destination’ tables.^1^ For each pair of years we cross-classify firms into their size-bands in year *t* – these are their origins – the rows of the table, against their size-bands in year (*t* − 1) – these are their destinations (one of which is ‘death’) – the columns of the table, and these annual tables allow us to chart the rhythm of the growth process. Complementing these annual tables, a cross-classification of size-band at birth against size-band at age 15 provides an effective tool for organizing the data on the growth trajectories of the 26 thousand survivors. Then, by plotting the ‘slope’ of the growth trajectories, we are able to reveal the evolution through time of a set of size-differentiated growth paths which exhibit a period of relative turbulence in the early years up to age five, followed by a decade long period of relatively stable growth (or in some cases decline).

The next section reviews the literature on firm growth, followed by a section describing data sources and construction. There are then five sections which make up the main body of the paper, and these,

- introduce the cohort of firms born in 1998 (cohort98) and summarise its performance over its first decade and a half of life
- report survival functions, for the cohort as a whole, and by size-band
- use an origin/destination matrix to investigate the connection between firm size at birth and at age 15 and growth over the period
- use a set of annual mobility tables to summarize the pace and direction of the change in jobs
- describe trajectories by size-band, and the ’slope’ of those trajectories and what the ’slope’ plots reveal about growth over the first 15 years of surviving firms’ lives

A final section sums up.

## The literature and method

### A sketch of the literature on firm growth

There are two distinct, but occasionally intersecting, strands in the literature on firm survival and growth which form the context for our investigation. One, with the longest history, has origins conventionally traced back to abook, published by Gibrat in 1931 as the source of ’Gibrat’s Law’: the proposition that afirm’s growth rate over aperiod is independent of it’s size at the beginning of the period. The empirical investigation of this proposition, and its implications for the firm size distribution, has inspired ahuge literature,^2^ much of which has been concerned with fitting increasingly exotic statistical distributions to the firm size distribution (see Axtell (2006)), and more recently the distribution of firm growth rates (see for example Bottazzi and Secchi (2003)) .One of the principal conclusions of amonograph which provided awide-ranging survey of empirical work on firm growth (and firm growth rates, and Gibrat’s Law) is of particular relevance:

“We wrap up by .. arguing in favour of Herbert Simon’s (1968) research strategy, which emphasizes the need for solid empirical work to first produce the ’stylized facts’ that theory can then attempt to explain. At this stage, we consider that research into the growth of firms could benefit greatly from gathering of statistical regularities and ‘stylized facts’.” Coad (2009, p. 148)

The other relevant literature is abody of work on firm growth much of which finds its inspiration in another ‘classic’ text: Penrose’s 1959 monograph: *The Theory of the Growth of the Firm*. This literature has been recently, and very ably, surveyed by Davidsson et al. (2010) and it emphasises understanding growth from the firm’s point of view. However, here too, just below the surface, there does appear to be acurrent of dissatisfaction with the state of the field, but Davidsson et al.make adetermined attempt to take a‘problems as challenges’ view.^3^ In the introduction to aspecial issue of *Entrepreneurship Theory and Practice* published in the same year the issue’s editors are considerably more blunt:

“Even though there has been sustained interest in [firm] growth for almost 50 years, relatively little is known about this phenomenon and much misunderstanding and confusion surrounds it.” Leitch et al. (2010, p. 249)

However the distinction made here between these two literatures – broadly, ‘economics’ and ‘management’ – should not be over-emphasised. For example acomparison of the references in the Davidsson et al. (2010) and Coad (2009) bibliographies reveals quite anumber of common citations, the difference is rather more amatter of emphasis. Indeed, arecent contribution by Stam (2010) to the discussion of the direction of research on firm growth (helpfully) characterises these two strands as “randomness” (Gibrat) and “strategy” (Penrose) and he concludes:

“At least two major issues deserve further attention in the future: how to deal with randomness *and* strategy (i.e. not the traditional dichotomy of randomness *or* strategy) in the explanation of firm growth, and what kind of growth (path) is to be explained.” Stam (2010, p. 132)

As will be explained below, our approach provides a framework which allows a role for both ‘randomness’ and ‘strategy’ as Stam recommends.

### Growth trajectories

Since our central concern is tracking firm growth paths by age and size, it seems natural to organise firm-level data into ‘birth cohorts’ as this allows us, quite straightforwardly, to keep track of the size distribution of survivors as the cohort matures. Although a cohort approach is not very commonly applied in studies of size, survival and growth using firm-level data, there is a strand of work which (since it investigated the post-entry performance of start-ups) has relied on the cohort as an organising principle. One notable proponent of this approach, though focusing more on job creation than growth trajectories, has been Kirchhoff.^4^Cabral and Mata (2003) is a significant and rather better known example from the ‘economics’ literature but with a focus on the evolution of the firm size distribution. There is also a literature, much of it associated with Michael Fritsch (for an early example see Fritsch and Weyh (2006) and more recently Schindele and Weyh (2011)), which uses cohort data to help distinguish the ‘direct’ and ‘indirect’ effects on job growth of new business formation.^5^

It is interesting to note that the paucity of studies using longitudinal data has been aparticular source of complaint in the ‘management’ strand of the literature. For example, Dobbs and Hamilton (2007) are quite stringent in their criticism of the reliance on cross-section data collected for relatively short time periods which is then used to model growth.^6^ This critique leads immediately to akey conclusion,

“As this paper has identified, growth does not occur as alinear progression but is rather fraught with fluctuation and periods of stagnation. Cross-sectional designs may be able to identify some of the concomitants of small business growth.The major recommendation of this paper is that researchers adopt longitudinal research designs that enable them to trace the growth path of small businesses to which we can then begin to map the learning processes that can explain the observed behaviour.” Dobbs and Hamilton (2007, pp 315–316)

Whilst there are, of course, studies which have used longitudinal firm-level data and do study growth trajectories they seem to be very rare. We have found only ahandful. The most notable is Garnsey et al. (2006) whose motivation is similar to ours: “Little evidence is available on the growth paths of firms over time.” Garnsey et al. (2006, p. 9). Little seems to have changed since 2006 because although Garnsey et al.is widely cited (considerably more than 100 times) very few of the citations actually look at growth trajectories (for four studies which do deploy similar methods to longitudinal trajectory data see Diambeidou and Gailly (2011), Hamilton (2012), Coad et al. (2013) and Brenner and Schimke (2014)). What most immediately distinguishes our study from Garnsey et al.is the scale of the data, their study covers about 400 firms drawn from three countries (UK, Germany and the Netherlands) over periods (in some case) up to age 10. But there is amore fundamental, methodological, difference:

“In our analysis, employment growth has been used for the construction of *growth episodes* and the operational definition of *turning points*. We converted firms’ growth over time from interval to nominal scales. These represented types of growth episodes experienced, according to rate of growth over that episode. Asequence of summarized growth episodes was used to depict turning points in growth paths.” Garnsey et al. (2006, pp. 11-12)

We focus instead on mapping average growth trajectories for (relatively large) groups of firms, the loss of fine detail is an almost inevitable consequence of working with 26,000 trajectories.

### the ‘new’ literature on firm growth paths

In arecent paper Coad et al. (2013) have re-cast the ‘randomness’ approach to firm growth (familiar from the Gibrat’s law literature on the firm size distribution) and applied it to ”firm growth paths” (FGPs) – what we (and Garnsey et al.) have called ‘growth trajectories’.

“The theoretical starting point is that firm growth is well-approximated by arandom walk, and that survival depends on the stock of resources at start-up or accumulated from post-entry growth. Growth and survival are hence closely related. We theorize the growth and survival of new businesses by referring to agambler playing agame of chance. To continue playing the game, the gambler needs resources which can either be derived from ”wins” or from his/her own sources at start-up. This theory is known as Gambler’s Ruin.” Coad et al. (2013, pp. 615-616)

They used time series of annual sales growth for around 6,000 UK businesses born in 2004 to track firms over the first five years after birth. The FGPs are constructed by dichotomising each firm’s performance each year as either above, or below, the median growth of their population of firms in that year. So each FGP consists of a string of length five with a two-state alphabet, growth (G) or decline (D). So for example a sequence might look like ‘GGGDG’.^7^

Whilst the data are not entirely consistent with the ‘hypotheses’ which they deduce from the Gambler’s Ruin model, their overall conclusion is that it provides areasonable starting point for building amore satisfactory theory of firm growth,

“We make the case that Gambler’s Ruin applies most clearly to the newest and smallest firms. It is valid to regard such enterprises as corks in the sea driven by arange of factors beyond their power to control. However, once such enterprises have established some credibility in the marketplace, and also some scale, then they gain more control over their operating environment. At that time it might be expected that there would be an improvement in our ability to explain and perhaps ultimately predict future growth and survival. Given the strong chance elements present we do not expect this to be achieved with ahigh degree of accuracy, but our work implies that this task would become more tractable as new firms increase in scale and size.” Coad et al. (2013, pp. 628–629)

This paper prompted a series of exchanges – Derbyshire and Garnsey (2014), Coad et al. (2015), Derbyshire and Garnsey (2015) – which, although ending somewhat inconclusively, did help to identify the extent and nature of the common ground between “randomness and strategy” (see Stam (2010) quoted above). By so doing it serves to produce a clearer understanding of some of the issues involved in the theory of firm growth. To see how, though, it is necessary to step back slightly to gain some perspective.

First some terminology which may help to clarify matters. Astochastic process describes aphenomenon that evolves over time (process) and involves arandom (stochastic) component. It can be characterised by periodicity of observations (discrete/continuous), state space (possible values) and the extent and nature of the randomness which shapes the time-dependence of realisations (see for example Lindsey (2004, Chapter 1) for an introductory treatment). The views of both parties to the Coad/Garnsey discussion would appear (without either using the term) to accept that firm growth is describable as astochastic process. Apparently the difference between them lies in the description of the ‘mechanism’ producing randomness,

“We therefore have two competing explanations for purportedly random outcomes in relation to entrepreneurship. The first is that it is an indeterminate process equivalent to gambling. The second is that it is adeterministic process involving the iterative matching of internal firm resources to external opportunities, requiring entrepreneurial skill and effort but subject to deterministic chaos rendering prediction impossible.” Derbyshire and Garnsey (2014, p. 11)

Although Derbyshire and Garnsey clearly believe that deterministic chaos can be sharply distinguished from the outcome of ‘gambling’, this may not be straightforward. Considerable efforts have been devoted over quite a number of years to detect chaotic behaviour in economic time series (broadly comparable in character to growth in a population of firms), indeed there still does not seem to be agreement about the appropriate test (see Faggini (2014) for a recent survey). Moreover, it has recently been argued that deterministic and indeterministic (stochastic) systems in very many cases may be observationally equivalent (see Werndl (2009)).

Now Coad et al. (2015) (and in the original substantive paper Coad et al. (2013)) refer to their contribution as an application of the “Theory of Gambler’s Ruin”. Again some terminological clarification may help. In the literature on stochastic processes ‘Gambler’s Ruin’ (typically referred to as a ‘problem’ rather than a ‘theory’) is (as Coad et al.) note a variety of (one dimensional) random walk, which is in turn a particular kind of markov chain.^8^ So if we re-cast a firm growth model as a markov chain defined on a discrete state space – growth, no change, decline – we can in, fact encompass both Derbyshire and Garnsey (2014) and Coad et al. (2015), within a unified framework. It is also worth noting that by talking about a *sequence* of categorical states both Coad et al. and Derbyshire and Garnsey have (albeit implicitly) agreed to talk about firm trajectories rather than growth *per se*. And, as we shall see, this re-description of their approach makes clear its connection to our own.

### The ‘stylized facts’ of firm survival and job creation and their implications for studying job growth

Whilst there seems to be very little agreed knowledge about firm growth, following on from the earliest studies of large firm-level datasets (for example Evans (1987) and Dunne et al. (1989)) it has been accepted that a large proportion of firms die quite soon after birth (typically around half are dead by age 5), and that the proportion dying each year declines quite quickly as firms age. Of course, the detail varies from place to place and from time to time (and from sector to sector) and since 2011 the OECD has included non-parametric (graphical) estimates of survival functions for various of its member states in its annual publication *Entrepreneurship at a Glance* (see OECD (2015, pp. 60–61) for the most recent data).^9^

There is also quite general agreement that survival depends positively on size, but opinion is not unanimous (for example, see the widely cited study Audretsch et al. (1999) which found no relationship). There has also been a much more limited discussion of the relative importance of ‘current’ size, as opposed to size at birth. Although this distinction was made quite early on (see Phillips and Kirchhoff (1989)) it has received relatively little attention (for two exceptions see Mata et al. (1995) and Esteve-Pérez and Mañez-Castillejo (2008)). This distinction is important because it represents a potential link between growth and survival: if larger firms are less likely to die, then growing larger can, of itself, improve the survival chances of a firm born small.

Finally, there has been a prolonged controversy (now stretching back more than 30 years^10^) about the relationship between firm size and job creation which is also relevant here. David Birch in a late 1970s consultant’s report (investigating the importance of firm migration in accounting for cross-regional variation in job creation) was somewhat surprised to find that a relatively small number of firms– disproportionately *small* firms – accounted for a relatively large proportion of job creation (for an accessible summary see Birch (1981)). Although Birch’s claim about the scale of the small firm contribution proved controversial (see for example Davis et al. (1996)), the broader conclusion became widely accepted quite quickly (see for example the discussion in Storey and Johnson (1987)), and interest in it continues.

Whilst the extensive literature on ‘job creation’ (as distinct from job growth) lies beyond our scope, there is one finding which recently emerged in Haltiwanger et al. (2013) which is directly relevant to the discussion of job growth trajectories. Specifically Haltiwanger et al.introduced aterm – whose use seems to have become quite widespread – to characterise newborn firm performance: “up-or-out dynamics”,

“The up-or-out pattern of young firms also helps put the job creation from start-ups in perspective. Each wave of firm start-ups creates asubstantial number of jobs. In the first years following entry, many start-ups fail ... but the surviving young businesses grow very fast.” Haltiwanger et al. (2013, p. 348)

Moreover, this characteristic of firm dynamics has awider significance, since it allows them to link the empirics of job creation to some well-known theoretical models of firm behaviour,

“This dynamic is an important feature of market-based economies and is consistent with predictions in models of market selection and learning (see Jovanovic, 1982; Hopenhayn, 1992; Ericson & Pakes 1995).” Haltiwanger et al. (2013, pp. 347–348)

Interestingly, in amore recent paper, Haltiwanger et al.seem to take amore nuanced position.

“Recent research shows that the job creating prowess of small firms in the U.S. is better attributed to startups and young firms that are small. But most startups and young firms either fail or don’t create jobs. Asmall proportion of young firms grow rapidly and they account for the long lasting contribution of startups to job growth.” Haltiwanger et al. (2015, abstract)

Perhaps this might be better encapsulated as ‘out or not up’ (since ‘not up or out’ is a little ambiguous). Certainly it does not seem to bear the same relationship to ‘models of market selection and learning’ as did the earlier statement.

However, there is abridge between the two formulations which Haltiwanger (and his co-authors) have helpfully provided,

“.. within the category of startups, we should expect to find various types of entrepreneurs. Schoar (2010) argues for distinguishing between ”subsistence” entrepreneurs and ”transformational” entrepreneurs. Her distinction was intended primarily for emerging economies where many entrepreneurs have limited prospects for growth, but we think this distinction is useful for the US economy as well. Subsistence entrepreneurs can be thought of as those that create small businesses that provide employment for the entrepreneur and perhaps afew others (often family members), which do not usually grow ... When people discuss the importance of entrepreneurs in job creation and productivity growth, they are envisioning transformational entrepreneurs, not subsistence entrepreneurs.” Decker et al. (2014, pp. 5-6)

So it seems it is these “transformational entrepreneurs”, *a very small proportion of the startup population*,^11^ which display ‘up or out dynamics’.^12^

We have looked in some detail at the job creation literature though it addresses the question of firm growth only indirectly because it seems, in recent years, to have begun to provide some more firmly empirically-based generalisations about firm performance than were found in the literature which looked directly at the question of firm growth. We have the evidence from Haltiwanger and various co-authors across a number of studies of United States data (and similar findings for a range of OECD members, see Calvino et al. (2015)). Additionally, there is evidence from a six nation cross-country study of a birth cohort of firms which found that a small proportion of the very smallest firms (born with less than five employees) grew very quickly and contributed disproportionately to job creation over their first decade of life (see Anyadike-Danes et al. (2015)).

In brief, the job creation literature points firstly to age, and then secondly size, as critical dimensions in any account of job creation, and thus to firm growth whilst, thirdly, recognising the importance of allowing for heterogeneity in the evolution of the growth trajectories of firms born in the same year with the same size at birth.

## Data sources and construction

We use the UK Business Structure Database^13^ (compiled by the Office for National Statistics)^14^ which records annual data on employees for the entire population of firms in the UK. This data is compiled from a series of annual ‘snapshots’ of the Inter-Departmental Business Register, an administrative database which captures information from a range of sources, amongst them VAT returns and employer Pay As You Earn (PAYE) tax and social security records. The unit of analysis is an “employer enterprise” – a business with at least one employee^15^ – which we refer to as a firm. Firms may comprise a number of distinct local units (establishments or plants) but our data refer to firm-level employee numbers.

We have linked together the annual ‘snapshots’ from the BSD using firm-level identifiers to form a longitudinal firm-level database for the UK and have devised algorithms to produce firm-level demographic markers for ‘birth’ and ‘death’. The birth of a firm is dated by the first appearance of non-zero employment and its death is treated symmetrically and dated by the disappearance of the last employee. Of course our firms, which are in fact ‘employer enterprises’ (following the EUROSTAT-OECD Manual see EUROSTAT-OECD (2007, pp. 75–76)), may have a ‘pre-history’ as enterprises *without* employees, but they are not ‘born’ into our birth cohort until they take on their first employee and, symmetrically, our employee enterprises may continue to (legally) exist even after death, but with no employees.^16^ Moreover, our data do not distinguish between *de novo* births and those which result from the break-up of an existing firm, similarly the data do not distinguish between the closure of a firm and its disappearance due to merger. Although the data start in 1997, firms alive in 1997 could have been born in any previous year, so the first birth year we can identify with certainty is 1998.

Firms are classified as either ’private’ or ’public’ sectors and we make this split using the classification by industrial sector. All employees in – public administration and defence; education; and health and social work – as public sector (SIC92^17^ sections L, M, N) – are classified as public sector. Of course, some firms in these sectors (in health and education for example) are private, and some firms in our private sector are government-owned, but ours is a reasonable approximation and ensures that most typically longer lived public sector entities (like schools and hospitals) do not distort our calculations. The dataset used in the analysis reported here includes *only* firms in the private sector.

## Getting to know cohort98

The basic facts of cohort98 can be summarised quite simply and are set out in Table 1. At birth there were 240 thousand firms and just over 1 million jobs, fifteen years later only 26 thousand firms remained alive with about 400 thousand jobs. So in just 15 years 213 thousand firms died and almost three quarters of a million jobs were lost. One in ten firms survived to 2013, but the number of jobs in the surviving firms more than doubled, so on average survivors did grow.

**Table 1:** Cohort98, firms and jobs summary, birth to age 15

	birth	survivors	age 15	summary statistics	
		at birth			
firms ’000	239.6	26.2	26.2	survival ratio (%)	10.9
jobs ’000	1123.7	163.4	394.9	net job creation ’000	231.5
jobs/firm	4.69	6.25	15.09	growth ratio	2.414

**Source:** calculated from the Longitudinal BSD, see Section 3 for a description

**Notes:**

1. ‘survival ratio’ is the ratio of firm numbers at age 15 to firm numbers at birth

2. ‘net job creation’ is the cohort jobs at age 15 less survivor jobs at birth

3. ‘growth ratio’ is the ratio of jobs/firm at age 15 to jobs/firm in survivors at birth

**Fig. 1:**
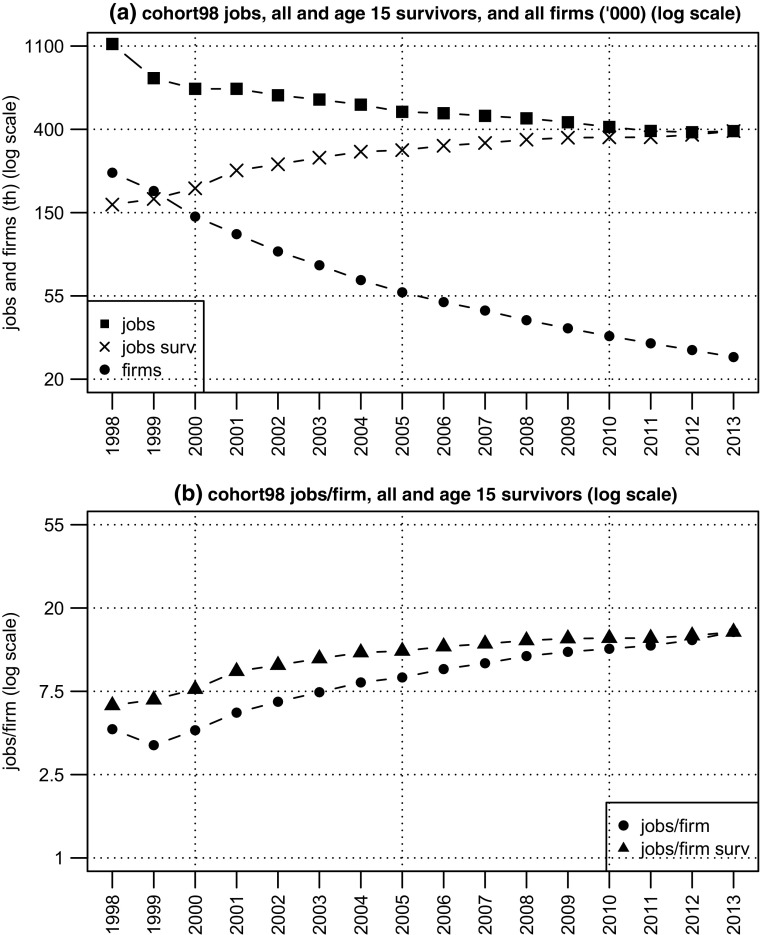
Cohort98: jobs and firms, birth to age 15 (log scale). **Source:** calculated from the Longitudinal BSD, see Section 3 for a description

The evolution of the cohort is plotted on panel (a) of Fig. 1 against a log scale so that the relative rates of decline in firm and job numbers are easier to see. Most of the loss of jobs occurred in the first five years (with a very steep drop in the year after birth). The rate of decline in firm numbers was even steeper, and continuous, but with a falling rate of decline. So, for example, between 1998 and 1999, 40 thousand firms died, whilst just two thousand were lost between 2012 and 2013. However the ‘raw’ cohort numbers give a rather misleading impression of the growth trajectories of the cohort’s firms. Looking back from the standpoint of 2013, we know that most of the jobs recorded in earlier years are jobs in firms which died. So if we are interested in the growth trajectories of 15-year old firms – their path from birth to age 15 – we are interested necessarily only in the jobs in the 26 thousand firms which survived. By 2013, the number of jobs in these survivors more than doubled from 163 thousand at birth to 395 thousand (by definition all of the cohort jobs in 2013 are in survivors), implying net job creation of almost a quarter of a million jobs. The growth path of survivor jobs is also plotted on panel (a) and we can see that it rises relatively smoothly, albeit at a declining rate, as it approaches 400 thousand in 2013.^18^

The path of jobs/firm is displayed on panel (b), and to ease comparability the tick marks have the same spacing here as in panel (a) (although the scales are different). After an initial dip (reflecting the large loss of jobs in the year after birth) the average size of firms expanded relatively smoothly, since jobs numbers were relatively stable and firm numbers fell. Since, by definition, the number of surviving firms is fixed, the jobs/firm ratio for survivors follows a path parallel to the survivor jobs series.^19^ The ‘growth ratio’ for 15 year old firms, computed as jobs/firm in 2013 divided by jobs/firm in 1998, is equal to 2.41 (equal in turn to the expansion ratio of the stock of survivor jobs) and implies an annual average growth rate of 6% in jobs/firm.

**Fig. 2:**
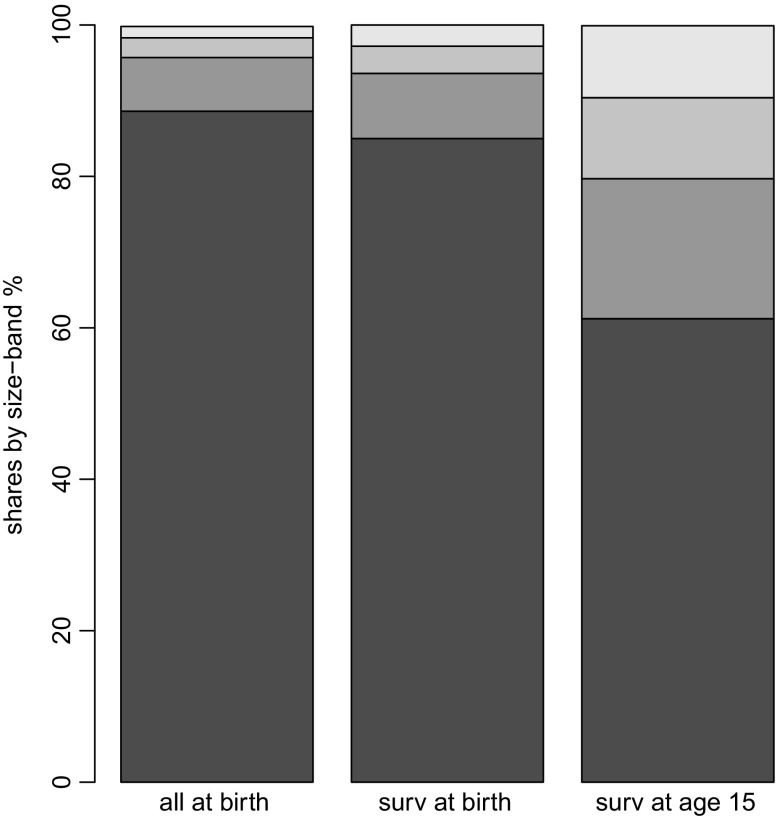
Cohort98:firm size distribution, birth and age 15, shares by size-band. **Source:** calculated from the Longitudinal BSD, see Section 3 for a description. **Notes:** 1. the two firm size distributions for age15 are: ‘surv at birth’ using birth size-bands; and ‘surv at age 15’, using age 15 size-bands; 2. the size-bands, from the bottom, are: ‘1–4’, ‘5–9’, ‘10–19’ and ‘20+’

To untangle survival and growth effects on the cohort as it ages we sacrifice some of the size detail and distinguish just four employee size-bands: 1 – 4; 5 – 9; 10 – 19; and 20+. The bars on Fig. 2 display the shares of firm numbers classified into size-bands. The first bar is is the cohort at birth, in its birth size-bands; the second bar is the age 15 survivors, also classified into their size-band at birth; the final bar also refers to the survivors at age 15, but now classified into their age 15 size-bands.

By comparing the bars we can separate the effects of survival – ‘birth’ vs ‘surv at birth’ – and growth – ‘surv at birth’ vs ‘surv at age15’. It is immediately obvious that differential survival effects play a much smaller role than differential growth effects in reshaping the firm size distribution. The share of firms in the smallest size-band is about 90% at birth, and is still 85% for the age 15 survivors at birth. By contrast, the shift in the survivor size distribution between birth and age 15 is quite dramatic. In particular, the share of the smallest firms, the 1 – 4 size-band, shrinks by around 25 percentage points, to about 60%. The shares in the larger size-bands all expand by (roughly) the same proportion: each of them have (at least) doubled in size. Whilst the death rate has had a huge impact on the overall size of the cohort (as we know by age 15 only 10% of the cohort remains) it is size-differentiated growth effects which have had much the larger impact on the firm size distribution.

## Survival by size-band

**Fig. 3:**
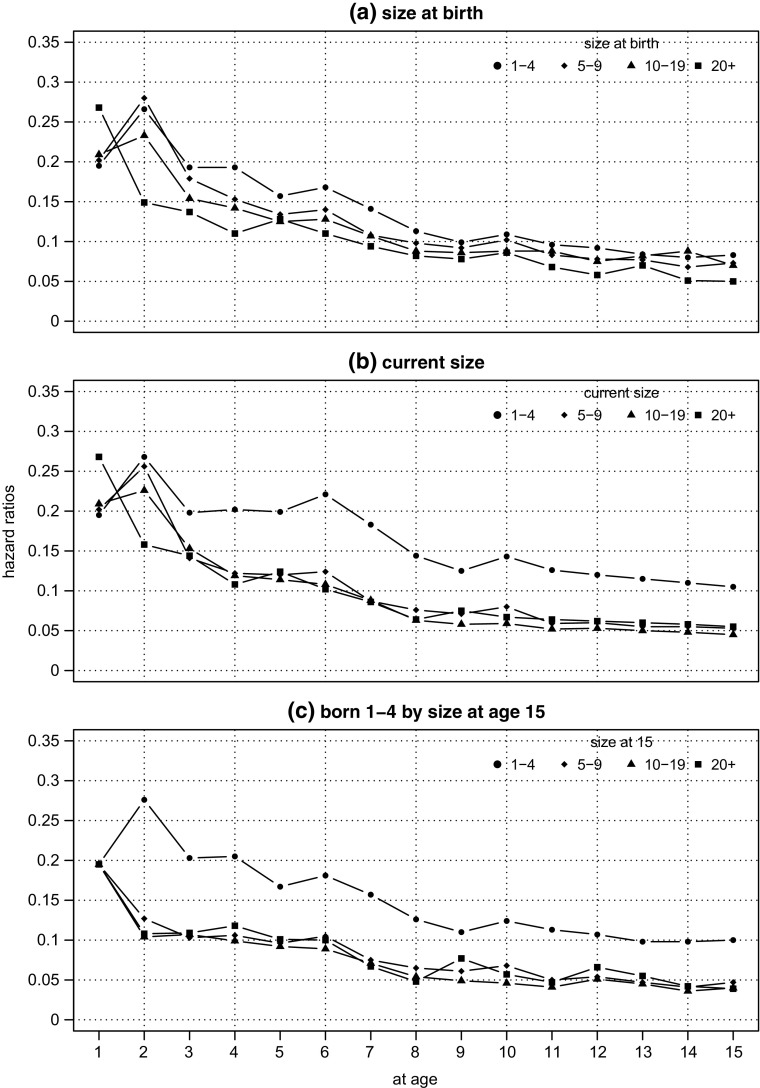
cohort98:hazard ratios by age and size-band. **Source:** calculated from the Longitudinal BSD, see Section 3 for a description

Before moving on to consider the growth trajectories of the cohort98 age 15 survivors, it is helpful to have a closer look at survival rates by size-band. Using the same four category size-band classification introduced in the last section we compute hazard rates – the proportion of those alive at age (*t* − 1) that are dead by age *t*. Figure 3 panel (a) displays the hazard rates computed for each year to age 15 by size-band at birth.

There is a striking contrast in the first year. The largest firms record a much larger hazard rate than the other three categories, but this pattern does not continue. The picture is transformed the following year, when the hazard for the largest firms drops quite steeply, and those for the other three size-bands spike upward. By age three the relationship between the four ratios settles into a pattern which then persists, with the hazard rates inversely proportional to size at birth: the smallest firms have the largest risk of death, the largest firms the smallest risk.^20^ The other obvious feature of the plot is the apparent convergence of the hazards: as all four populations shrink, their hazard rates become much harder to distinguish. However size-band related differences do remain. For example, at age 15 the hazard for the 1 – 4 size-band is 0.083, two thirds larger than the 20+ size-band which is 0.050 (the other two are about 40% larger: the 5 – 9 hazard is 0.073 and the 10 – 19 is 0.070).

Whilst *a priori* it may seem sensible to compute hazards by size-band at birth, there is an alternative approach which is not only plausible, but turns out to be quite revealing. By classifying firms into their size-band in the year immediately preceding their death, we can also compute hazard rates by ‘current’ size-band.^21^ In practice this means as firms grow (or decline) we continuously re-classify them, year by year, from one size-band to another. Of course any firm which very little positive or negative growth will remain in its birth size-band.

The process of re-classification as firms move between size-bands will have a limited impact in the early years, since firms will not have have had much opportunity to grow or shrink. However, we see from Fig. 3 panel (b), from age 4 onwards the picture begins to look quite different. Specifically, the hazard rate for the smallest firms declines much more slowly and, even more strikingly, a relatively wide gap opens up between the hazards for the smallest firms and the others which, in turn, become difficult to distinguish from each other. By age 15 the hazard rate for 20+ firms computed using the ‘current’ size band is very similar to its counterpart birth size-band rate, but the hazard for the smallest size-band is twice the ‘20+ rate’ and a quarter higher than the rate for 1 – 4 size-band at birth. Evidently, as smaller firms grow, and in particular as they grow beyond five employees, their survival prospects improve markedly. There is some improvement too in the survival prospects for the firms born in the 5 – 9 and 10–19 size-bands too, but not nearly as dramatic as for the smallest firms.

Panel (c) The bottom panel of Fig. 3 provides a more fine-grained picture of the ‘current’ hazard rate but just for firms born 1 – 4. It has been constructed by un-packing the figures for the larger size-bands and computing hazards for firms born 1 – 4 but which have moved into a larger size-band before death. So for example, there were 4,170 firms in the 5 – 9 age at age 14 which had been born in the 1 – 4 size-band, of these 197 were dead by age 15. So the hazard rate plotted at age 15 for 5 – 9 firms on Fig. 3 panel (c) is 0.047 (197÷4170). Of course, the 1 – 4 size-band line is the same as in panel (b): firms born 1 – 4 that were 1 – 4 in the year prior to death. Clearly then the effect we noted from panel (b), that growth improves the survival chances of the smallest firms is further reinforced. Indeed, comparing panels (b) and (c), we can see that (apart from the first few years) the hazards of the larger firms are virtually identical.

This finding is, in fact, a side-effect of the overwhelming importance of the 1 – 4 size-band. Even though, as we shall soon see, only a small proportion of these firms ‘migrate’ to larger size-bands, a small proportion of a very large number is sufficient to ensure that these in-migrants are a large proportion of the larger size-bands. This is our first evidence that growth (as opposed to size) affects the chance of survival but is the relationship symmetric? Is the hazard rate of the firms which move to the 1 – 4 size-band, for example, different from the hazard faced by firms which were born, and remained, small? It is not possible to answer this question with any precision because relatively few firms born in each of the larger size-bands die after having shrunk into size-band 1 – 4. However, if we average across all the larger size-bands, we can compute the hazard for all firms born with more than five employees which die after having shrunk into the 1 – 4 size-band. From age 5 onwards the hazard for firms born with *more* than 5 employees is indistinguishable from the corresponding hazard for firms born with *less* than 5 employees, following it down from around 0.150 at age 5 to 0.100 at age 15.

The two different hazard rate measures considered here – one conditioned on the size-band at birth, the other on the ‘current’ size-band – are associated with differently constructed origin/destination matrices. In the former the birth size-band provides the origin rows, in the latter the origin rows are the current size-band. As we see in the next two sections, both constructs can help inform the discussion of growth performance: the first captures in a single snapshot the movement across the size distribution over 15 years; whilst tracking the age 15 survivors year-by-year (so using the ‘current’ size-band) provides an insight into the evolution of the firm size distribution and a summary view of the pace of change, complementing the results on the ‘current size’ hazard rate.

## From birth to age 15

At age 15 there are 26,162 survivors whose distribution by size-band is recorded in Table 2 which has as its origin (rows) size-bands at birth in 1998, and as its destination columns size-bands at age 15 in 2013. Panel (a) displays data on number of firms, whilst panel (b) of the table expresses the cell counts as shares of the total. So these origin/destination tables ‘map’ firms by size-band at birth directly into firms by age 15 size-band. The entries above the diagonal are firms which have moved up a size-band, and those below have moved down a size-band, whilst those on the leading diagonal are, at age 15, in their birth size-band. Notice also from panel (b) that the shares of firms by size-band at birth in the ‘all’ column, correspond to those displayed on the middle (‘surv at birth’) bar on Fig. 2, whilst the shares by size-band computed from the ‘all’ row correspond to those on the right hand (‘surv at age 15’) bar.

**Table 2:** Cohort98 age 15 survivors, Origin/Destination matrix by size-band, firms and net job creation, birth (rows) vs age 15 (columns)

	1–4	5–9	10–19	20+	all
(a)firms					
age 15 survivors					
1-4	15011	3973	1997	1248	22229
5-9	721	642	489	407	2259
10-19	196	180	226	334	936
20+	84	55	91	508	738
all	16012	4850	2803	2497	26162
(b) firms age 15					
shares of all (%)					
1-4	57.4	15.2	7.6	4.8	85.0
5-9	2.8	2.5	1.9	1.6	8.6
10-19	0.7	0.7	0.9	1.3	3.6
20+	0.3	0.2	0.3	1.9	2.8
all	61.2	18.5	10.7	9.5	100.0
(c) net job creation					
’000					
1-4	6.5	18.6	22.7	91.6	139.3
5-9	-2.8	0.3	3.0	23.4	24.3
10-19	-2.1	-1.0	0.2	30.5	27.7
20+	-5.8	-1.8	-6.8	54.4	40.1
all	-4.2	16.1	19.6	199.9	231.5

**Source:** calculated from the Longitudinal BSD, see Section 3 for a description

**Notes:**

1. panel (b) is panel (a) ÷ 26,162, expressed as a percentage

2. ‘net job creation’ is the cohort jobs at age 15 less survivor jobs at birth

The table has a number of noteworthy features. Firstly, most firms born 1 – 4 are in the 1 – 4 size-band at age 15: these are the 15,011 firms in the cell in the top left hand corner of panel (a) – almost 60% of the total.^22^ The entries on the ‘leading diagonal’ of the matrix for the 5 – 9 and the 10 – 19 size-bands are not the largest entries in their rows, nonetheless these firms too are quite likely to be in their size-band of birth at age 15. Secondly, entries in the table above the leading diagonal are always larger than the entries below. For example, many more firms born 1 – 4 grow into 5 – 9 (3,973) than do 5 – 9 born firms shrink into 1 – 4 (721). Finally, and this is perhaps the most striking observation: the largest ‘origin’ size-band for firms in the 20+ ‘destination’ size-band – *by a very wide margin* – are those born with less than five employees, indeed almost exactly half of all 20+ firms at age 15 were born in the 1 – 4 size-band.

Table 2 panel (c) gives a first indication of the significance of firm mobility for the growth in jobs. Again it is an origin/destination table classifying size-band at birth against size-band at age 15, but in this case the entries are ‘net job creation’: the cell by cell difference between jobs at birth and jobs at age 15. By construction, of course, entries above the leading diagonal are necessarily positive – firms moving up a size-band must have added jobs. Equally, entries below the leading diagonal are necessarily negative – firms moving down a size-band must have lost jobs. Net job creation by firms in cells on the leading diagonal could in principle be positive or negative – since firms can gain or lose jobs and remain in the same size-band – but here they are all positive.

This table provides some perspective on a long-running argument about the connection between firm size and job creation. If we classify firms according to size at birth, then the row sums in the ‘all’ column measure the birth size-band contribution to net job creation. The principal feature of this data is quite clear: firms born into the smallest size-band contribute more than half of net job creation. However, as we know (from Fig. 2 and panel (b)) these firms account for about 85% of survivors, so a sizeable contribution might have been expected. If instead we use the ‘all’ row – classifying contributions by firm size at age 15 – the picture looks radically different. Although firms in the 1 – 4 size-band *at age 15* account for almost two thirds of the survivor population, their combined contribution to net job creation is negative. Clearly, measurement conventions matter a good deal in this case.^23^

The most striking entry on panel (c) of Table 2, though, is in the top right hand corner – it records the number of jobs created by the 1,248 firms which were born 1 – 4, and which by age 15 grew to be 20+. We can see that these firms account for 91.6 thousand of the total 231.5 thousand of (net) jobs created by all cohort98 firms. Of course, this calculation has to be carefully interpreted because, as we can see, there are positive and negative numbers entering into the overall total. Nonetheless, it is a striking finding that this relatively small group of firms – less than 5% of survivors (about 0.5% of the cohort at birth) – make such a huge contribution to net job creation.

Table 3 presents a further set of origin/destination tables. These summarise data on jobs/firm for the 15 year old survivors: panel (a) displays the figures for survivors at birth; panel (b) jobs/firm 15 years later; and panel (c) records the growth ratio, the ratio between (b) and (a). We have already seen some of these numbers before, the bottom right hand cell in each table – at the intersection of the ‘all’ row and the ‘all’ column – in the jobs/firm row of Table 1. What we can see immediately from panel (a) of Table 3 is that firms in the cells above the leading diagonal (i.e. firms which move up the size-band distribution) were in every case larger at birth than firms which remain in their size-band at birth. In summary: they start larger (although not always by much) and end larger.^24^

**Table 3:** Cohort98 age 15 survivors, Origin/Destination matrix by size-band, jobs/firm, birth (rows) vs age 15 (columns)

	1–4	5–9	10–19	20+	all
(a) birth					
1-4	1.50	1.84	1.92	1.96	1.63
5-9	6.21	6.29	6.54	6.60	6.37
10-19	12.81	12.45	12.92	13.54	13.03
20+	70.19	39.58	89.22	166.25	136.38
all	2.21	3.25	6.44	37.69	6.25
(b) age 15					
1-4	1.93	6.52	13.3	75.33	7.99
5-9	2.35	6.75	13.49	64.07	17.13
10-19	2.15	7.09	14.01	104.98	42.65
20+	1.68	7.27	14.97	273.36	190.74
all	1.95	6.58	13.45	117.75	15.09
(c) growth ratio					
1-4	1.29	3.54	6.93	38.43	4.85
5-9	0.38	1.07	2.06	9.71	2.69
10-19	0.17	0.57	1.08	7.75	3.27
20+	0.02	0.18	0.17	1.64	1.40
all	0.88	2.02	2.09	3.12	2.41

**Source:** Calculated from the Longitudinal BSD, see Section 3 for a description

**Note:** ‘growth ratio’ is the ratio of jobs/firm at age 15 to jobs/firm in survivors at birth

The most revealing of the panels in Table 3, though, is panel (c): the growth ratios. Of course the general pattern is as might have been anticipated. Firms in cells above the diagonal – those which move up – grew faster than those on the diagonal; and firms in cells below the diagonal – those which moved down – grew slower than those on the diagonal. As with the net job creation table, there is an interesting contrast between the ‘all’ column and the ‘all’ row. If we were to use the ‘row measure’ of growth over 15 years, classifying firms by their size-band at age 15, the conclusion would be that larger firms grow faster than smaller: 20+ firms recorded more than three times(3.12÷0.88) more growth than firms in the 1 – 4 size-band. In strong contrast, the conclusion from the classification based on size-band at birth, the ‘column measure’, would be the reverse: small firms grow faster than larger firms, the average for firms born 1 – 4 is 3.5 times (4.85÷1.40) that of firms born 20+.

The most spectacular, and noteworthy, entries (unsurprisingly) are those for firms which grow into the 20+ size-band. For our 1,248 exceptional firms born with less than five employees the growth ratio is 38.43, implying an annual average growth rate of about 30%, their average size expanding from just under two jobs/firm at birth to 75, 15 years later. By contrast, the rates of expansion of firms born 5 – 9 and 10 – 19, also recorded in this column, are much more modest, implying annual average growth rates of around 15%. As we know the 20+ entry in the 20+ column covers all the firms born in this size-band whose employee numbers were still above 20 at age 15 years. So it includes firms which grew, firms which did not, and firms which shrank (to size 20). On average this group did grow, but very slowly, at not quite two thirds of the cohort average rate. It is also worth noting the degree of ‘shrinkage’ in firm size in the 20+ row. For example, there are 84 firms born with (on average) 70 jobs per firm which had by age 15 less than 2 jobs: so about 2% of their birth size.

## Year-to-year mobility

As a first step in describing the process of change we have constructed a series of year-to-year origin/destination tables for the 26,162 age 15 survivor firms, with one table for each pair of years from birth to age 15: one for birth to age 1; another from age 1 to age 2; and so on, through to age 14 to age 15. The simplest way to display the process of change depicted in these 15 tables is first to convert the tables into row-standardised (‘markov’) form – each entry (origin/destination pair) in a table is expressed as a proportion of its corresponding row total – so each entry will record the proportion of the number in a size-band (row) in year *t* which move into each size-band (column) in year (*t* + 1).^25^ In effect we are providing a description of the year-to-year process of size-band mobility which connects the size-band distribution at birth with the size-band distribution at age 15. In other words, we are describing the pattern of change which turns the bar labelled ‘surv at birth’ on Fig. 2 into the bar labelled ‘surv at age 15’ (or, to put it differently, the year-by-year evolution of the rows of panel (a) of Table 2 into the columns of the table).

**Fig. 4:**
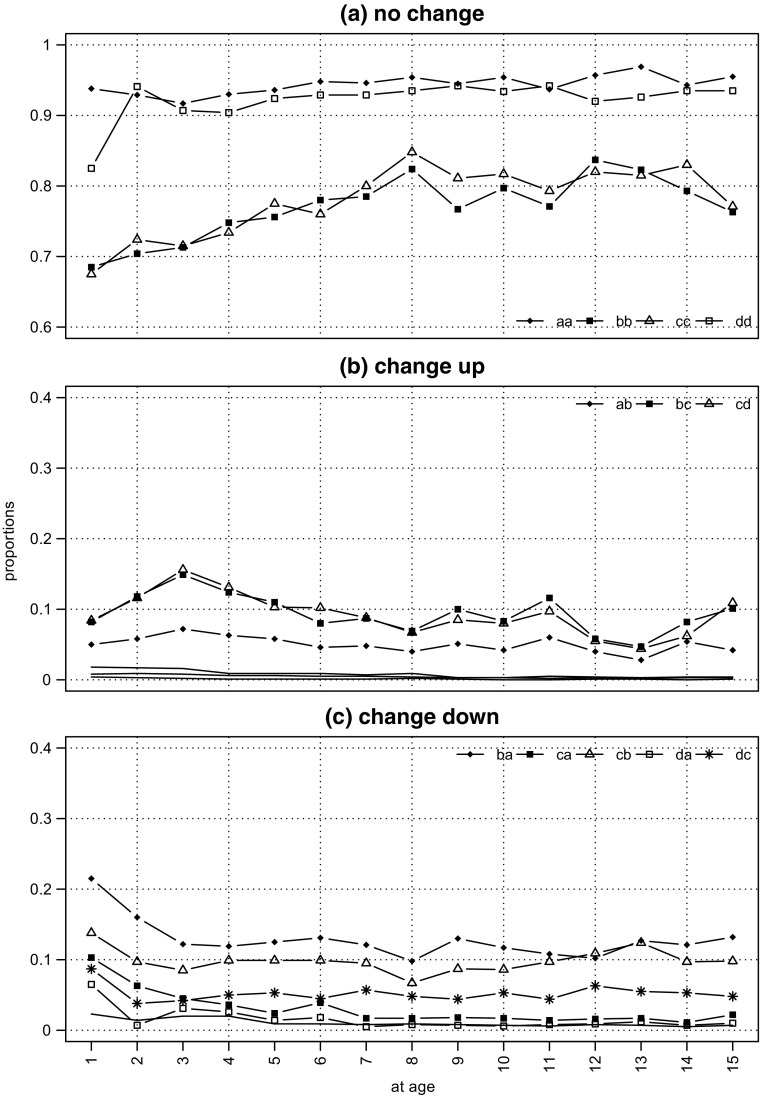
Cohort98 survivors to age 15: year-to-year-size-band mobility. **Source:** calculated from the Longitudinal BSD, see Section 3 for a description. **Notes**: 1. the size-bands are: “a”, ‘1–4’; “b”, ‘5–9’; “c”, ‘10–19’; and “d”, ‘20+’2. the first letter of a pair is the origin size-band, the second is the destination size-band; so “aa” is the proportion from “a” in *t* which is in “a” at *t* + 1

We can distinguish three ‘types’ of (row-standardised) proportions: ‘no change’ entries – the leading diagonals of the origin/destination matrices – the proportion of firms which remain in the same size-band from year *t* to year (*t* + 1); ‘up change’ entries – the entries above the leading diagonal – the proportion of firms which move up a size-band from year *t* to year (*t* + 1); and ‘down change’ entries – the entries below the leading diagonal – the proportion of firms which move down a size-band from year *t* to year (*t* + 1). All the proportions are displayed as annual time series on Fig. 4: ‘no change’ on panel (a); ‘up change’ on panel (b), and ‘down change’ on panel (c). Finally, each series is labelled by an alphabetic pair, the first letter in the pair indicates origin size-band (at age *t*), and the second letter the destination size-band (at age (*t* + 1)), the key is set out below the plot. So for example, on panel (a), the first ‘no change’ series ‘aa’ is the proportion from size-band ‘a’ at age *t* which is in size-band ‘a’ at age (*t* + 1); on panel (b) the first ‘up change’ series ‘ab’ is the proportion from size-band ‘a’ at age *t* which is in size-band ‘b’ at age (*t* + 1); and on panel (c) the first ‘down change’ series ‘ba’ is the proportion from size-band at ‘b’ age *t* which is in size-band ‘b’ at age (*t* + 1). To simplify comparisons between the panels, they each have tick marks at intervals of 0.05 (even though the range of the scales differ). The axis across the bottom records the ‘destination’ year: so, for example, year 1 is the transition proportions from birth to year 1.

Inspection of the three panels of Fig. 4 reveals that by far the largest proportion of firms in each size-band is in the ‘no change’ data category displayed on panel (a). For the smallest and the largest size-bands the proportion fluctuates within a very narrow range, typically between 90% and 95% for virtually the entire period. For the intermediate size-bands there are two quite distinct periods, from birth to about age 7 the proportion rises from just below 70% to 80%, from age 7 onwards it fluctuates around 80%.

The ‘no change’ proportions for the 5 – 9 and 10 – 19 size-bands look quite different from those for the largest and smallest size-bands in two distinct respects: first they seem to change quite systematically as the cohort ages; second, the proportions are always 20 to 30 percentage points lower. From birth, up to about age 5 or 6, for both the mid-size groups, the ’no change’ proportion steadily rises (at a rate of about two percentage points a year), after which it seems to stabilise in the 75% to 85% range. Looking down to the lower panels we can see that the proportions moving down are relatively large (but declining) whilst the proportion moving up are relatively small (but rising). However, in both cases, the proportions are only large for movements into ’nearest neighbours’ – down to 1 – 4 and up to 10 – 19 for the 5 – 9 size-band; down to 5 – 9 and up to 20+ for the 10 – 19 size-band. This early turbulence might perhaps be interpreted as evidence for an initial, ‘churning’, phase in the evolution of the survivor firm size distribution. Indeed the distinct ‘spike’ in the hazard functions at age 2 we saw earlier could well be part of the same process.

We can draw a number of conclusions about the year-to-year pace of change over the cohort’s first 15 years. First, ‘no change’ is always very much the most likely.^26^ Secondly, of the relatively small proportion of firms which do change size-band, very, very few move further in one step than their nearest neighbour size-band. Thirdly, there is a clearly identifiable ’shake-out’ period in the first five or so years of life when there is rather more movement of firms both up and down the size-band distribution.^27^ Finally, it is important to recognise that, even though there is movement both ‘up’ and ‘down’ the size-band distribution in every year, the ultimate effect of this re-shuffling is to produce by age 15 a firm size distribution with a much smaller share of firms in the 1 – 4 size-band and correspondingly larger shares in the others. This, after all, is the clear message from Fig. 2. So we know that although most movement year-to-year is no further than the nearest neighbour, by the end of the period there has been a systematic shift , and this raises two questions. Looking back from the vantage point of age 15: what proportion of firms have left their birth size-bands? and how far have they gone?

Of course, we already know how to find the answers: from Table 2, the birth to age 15 orgin/destination table. For example, of the 22,229 surviving firms which were born 1 – 4, 15,011 were in 1– 4 at age 15, more than half the seven thousand ‘movers’ were in the 5 – 9 size bands, another quarter had moved up two size-bands to 10 – 19, with the remaining 1,248 (less than 20% of movers) in the 20+ size-band. Whilst the proportions remaining in the same size-band after 15 years are very considerably smaller than in the annual tables, the overall pattern remain qualitatively similar: there is a concentration on the leading diagonal, and much of the movement is to nearest neighbours, it declines steeply with ‘distance’.

## Growth trajectories

### The trajectories

**Fig. 5:**
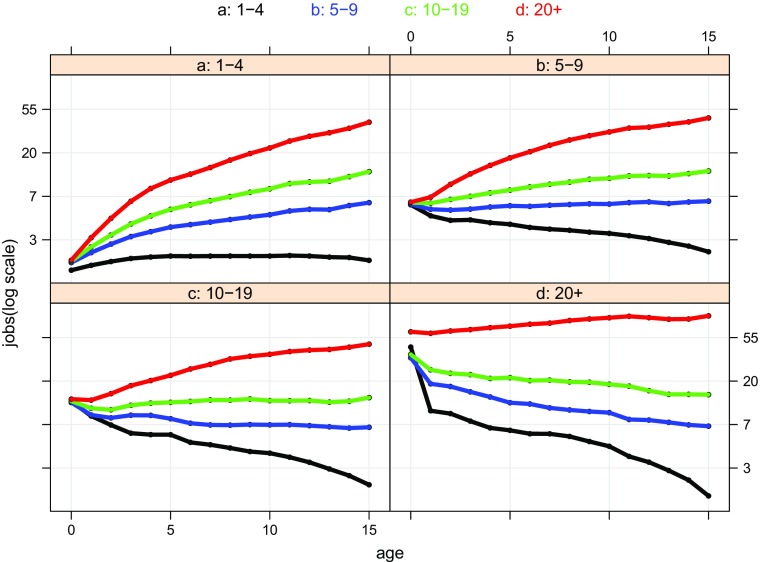
Cohort98 survivors to age 15: jobs/firm by age trajectories, size-band at birth and age 15 (log scale). **Source:** calculated from the Longitudinal BSD, see Section 3 for a description. **Note:** panels are size-bands at birth, within each panel the colour-coded lines denote size-band at age 15, the key is above the display

Having cleared the ground with an investigation of survival and the overview of mobility, we now turn to characterising the growth trajectories of cohort98’s 15 year old survivors. The challenge is to provide an interpretable summary of all 26,162 trajectories. The empirical strategy adopted here is to describe these trajectories by making use of our four size-bands. Cross-classifying firms by size-band at birth and by size-band at age 15 yields the four–by–four classification into 16 different groups of firms corresponding to the cells of the origin/destination matrix of Table 2. Figure 5 displays the average job/firm ratio^28^ for each group, and the time series have been plotted against a log scale, so that the slope of the curve can be interpreted as a rate of growth. The 16 groups are organised into four plots, by size-band at birth, and within each plot the trajectories are colour-coded according to their size-band at age 15: 1 – 4, black; 5 – 9, blue; 10 – 19, green; and 20+, red. What we have in effect is a graphical rendering of a sequence of origin/destination tables, where each plot corresponds to a row of the origin/destination table recording annual observations on each column. The number of firms included in each averaged trajectory is, of course, the numbers recorded in the corresponding cells in panel (a) of Table 2.

The ‘big picture’ reveals a striking degree of regularity. Within a couple of years of birth the paths heading to the different age 15 destinations are quite distinct with the expected ranking: the red curves – firms heading to 20+ are at the top; firms heading for 1 – 4, coloured black, are at the bottom. Looking in more detail at the top left hand panel of Fig. 5, the growth trajectory coloured in red is that of that familiar group of 1,248 firms which are born 1 – 4 and which have more than 20 employees by age 15. The curve rises very steeply (albeit at quite a steeply declining rate) up to age 5, beyond which it becomes a more-or-less straight (but still upward sloping) line. So there is a very rapid take-off, after which growth slows, and then becomes constant. Looking at the comparable – red-coloured – growth trajectories for firms born 5 – 9 and 10 – 19, we see a similar pattern: relatively rapid growth in the early years which slackens, and then steadies as firms age. The firms born 20+ and remaining 20+, in the bottom right hand panel, are a conceptually different group, since (as we know) they are a mixture of firms which grow and those that do not. Taken together, perhaps unsurprisingly, these largest firms exhibit (on average) very little growth. There is some similarity in the trajectories of the different groups of firms headed for size-band 1 – 4. The sharp contraction in firms born 20+ – the black curve in the bottom right hand panel – is quite striking. What does seem to differentiate the contracting groups, though, is that the rate of contraction, after having moderated, seems to increase again as the firms approach age 15. The growth trajectories of the intermediate groups which involve rather less dramatic expansion or moderate contraction, essentially the blue and green curves, look, typically, rather smoother, except in the first few years. But the precise patterns are difficult to discern from the plots of the trajectories themselves, we need a sharper picture which a plot of the slopes of the trajectories turns out to provide.

### Trajectory slopes

**Fig. 6:**
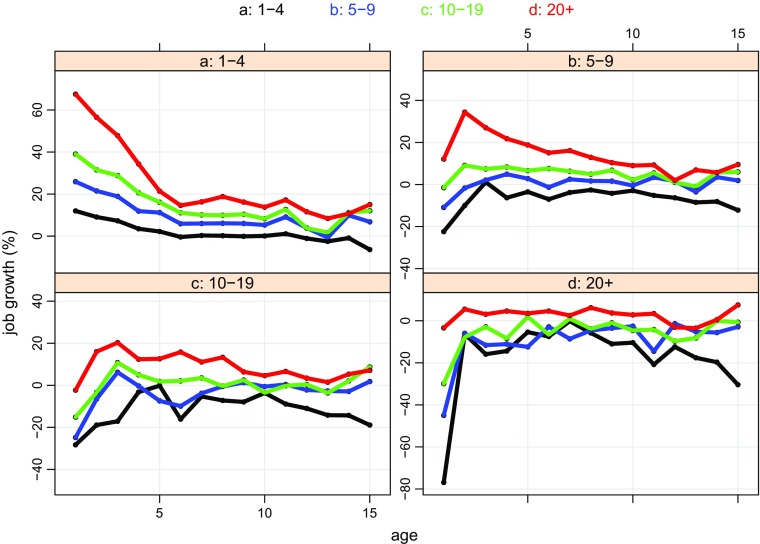
Cohort98 survivors to age 15: jobs/firm by age slope of trajectories, size-band at birth and age 15 (log scale). **Source:** calculated from the Longitudinal BSD, see Section 3 for a description. **Note:** panels are size-bands at birth, within each panel the colour-coded lines denote size-band at age 15, the key is above the display

Figure 6 displays a plot of the *‘slopes’* of the trajectories of the jobs/firm ratios^29^ which have been organised into the same four panels by size-band at birth and, again, within each panel the curves are colour-coded according to size-band at age 15 (the assignment of colours to size-bands remains the same). Although the scales vary across panels, the distance between the maximum and the minimum on each panel is the same and there is a common distance of 20 percentage points between tick marks. The benefit of this common distance is immediately obvious: we can see that, after age 5, most of the growth rates fall within a relatively narrow 20 percentage point range. For the three smaller size-bands it is 0 to 20%, whilst for the 20+ size-band it is 0 to -20%. There is though a distinct ‘dip’ in most growth rates after age 11 – this is 2009, so it may be associated with the ‘Great Recession’ – but most rates recover by age 14. Finally, it is worth noticing that some of the negative growth rates recorded by firms contracting into the 1 – 4 size-band by age 15 are exceptions to this ‘later life’ generalisation with observations falling outside a panel’s common 20 percentage band particularly after age 10.

The pattern of growth in the first five years also yields some interesting generalisations. The smallest firms expanding to 20+ (the red curve in the top left hand panel) display a very steep take-off, about 70% from birth to age 1, but this growth drops about 10 percentage points a year until age 6, when it flattens out at about 20% a year. The next two size-bands, 5 – 9 and 10 -19, as they grow to 20+ look a little different, in both cases, the maximum growth occurs a little later (age 2 and age 3 respectively), before growth slows (as noted above growth for the group born 20+ is much more muted and largely featureless). Initially the pattern of contraction by firms heading towards the 1 – 4 size-band (the black curve) is, broadly, a mirror image of that of expansion of firms heading towards 20+. The most dramatic adjustment is by firms born into the largest size-band – they record a contraction of 80% in their first year, after which the rate falls to around 10% a year. Similarly, the 10 – 19 and 5 – 9 size-bands record very large rates of contraction in the first few years. Where contracting firms differ from those expanding, though, is the pattern after after age 10: all the larger size-bands display increasing rates of contraction, so the curves appear as slightly concave to the age axis.

Looking back to Fig. 4, we can see how the changing proportions in the annual mobility plots reflect the evolution of growth rates. For example, the differing ‘time-shapes’ of expansion and contraction can be linked to the contrast in the ‘time-shapes’ of the proportions moving up and down the size-band distribution. Equally, we can see how the initial spurts in expansion and contraction and the relative quiescence after age 5 fits with the initial ‘turbulence’ in proportions.

## What have we learned?

### Findings

Each year a new cohort of firms is born, each year a proportion of the firms from previous cohorts which had, so far, survived will die. These two – birth and death – are the drivers of change as the population of firms evolves. In the UK there were about a quarter of a million of firms in the 1998 birth cohort of private sector firms. The vast bulk of them were very small (just 1 – 4 jobs), most of the firms which subsequently died were small too, both because there were so many more of them, and because very small firms have a lower chance of surviving. However, as they age, firms’ chances of survival improve. The growth of firms, like their survival, depends on age and size too, but the relationship between growth and age and size is the reverse of that for survival. Young firms are more likely to grow than older firms, and smaller firms which grow are more likely to grow at a faster rate than older firms which grow. Here, to make the data analysis more tractable, we have used size-bands, to discuss survival and growth. This has the benefit of dampening (to some extent) the extraordinary individual-level heterogeneity found in a huge collection of firm-level records and, of considerable practical importance, it has helped to ensure that we can report some noteworthy findings whilst still complying with the disclosure control requirements of the statistical authorities.

Although 10% of firms born with less than five employees survived to age 15, for firms born with more than 20 employees the proportion was 20%, twice as large. However, by age 15, the hazard rates – the risk of dying in the next year – for these two groups of firms had become quite similar at about (8% for less than five, 5% for more than 20). More revealing, though, than the comparison of hazard rates by size at birth are hazard rates by ’current’ size-band, that is the hazard rates computed using the size-band classification for firms in the year before death. For current size-bands the hazard rate for firms *still* very small remains relatively high, at 11%, whilst for firms *no longer* very small, whether born very small or not, it is considerably lower, at 5.5% it is about half the hazard rate of the smallest firms. Evidently, growing out of the smallest size-band substantially improves a very small firm’s chances of survival. Equally, it is evident that shrinking into the smallest size-band is associated with a clear worsening of a firm’s survival prospects.

Our investigation of firm size-band mobility revealed that, certainly after age five, there was considerably less year-to-year mobility, and most firms that did move size-bands did not move (in one annual step) much further than their nearest neighbouring size-band. Taking the longer view, cross-classifying firms by size-band at birth and by size-band at age 15, we can see the cumulated effect of change. Whilst inertia is still clearly evident in the firm size distribution, especially at the upper and lower ends, the sheer importance of very small firms in the cohort at birth produces, even without much mobility, a relatively large number of firms born with 1 – 4 jobs which exhibit substantial growth and make a correspondingly substantial contribution to the cohort’s job creation performance. The surviving born large firms also add importantly to the cohort’s job creation performance, even though they record quite modest rates of growth.

Whilst birth to age 15 comparisons are suggestive, and annual mobility can shed light on the pace of change, it is only by investigating growth trajectories – the 15 year job histories - of cohort98’s 26 thousand survivors that we are able to properly differentiate the growth paths of different groups of firms. We find that in each of our four size-bands there are varying proportions of firms which, grow or shrink or show no growth at all. Our examination of the changing ‘slope’ of the growth trajectories finds a degree of regularity in the pattern of change over time. Across all the trajectories we find that most of the largest changes occur in the period up to age five. After the initial relatively turbulent phase, beyond age 5, and even more obviously beyond age 10, expansion rates seem to settle down and appear to fluctuate between rather narrower bounds, there is though some evidence that contraction rates may begin to accelerate after age 10.

### Implications for ‘theory’ and ‘practice’

First it is important to be clear about what might be expected of a ‘theory’ of firm growth. Since it now seems quite widely accepted in the epistemological literature that the theory/model distinction may not be tenable^30^ we may be permitted to argue that there may be no important difference in principle (let alone practice) between a theory of firm growth and a model of firm growth. Given this first step, we can then ask how a model of firm growth might be characterised? We have seen that Coad et al. concede ‘Gambler’s Ruin’ is a first step: “which applies most clearly to the newest and smallest firms.” Coad et al. (2013, p. 628). Indeed, it is our contention that a random walk is rather too limiting as an account of even the early years of firm growth, and that a rather ‘richer’ stochastic process may better account for the substantial heterogeneity in firm performance. Here we propose as an alternative starting point a non-stationary first order markov chain with size-band dependent transitions. Of course, some of the characteristics we report might be be specific to the UK, or even to this cohort. Moreover, choices like size-band width may make a difference to the structure of the transition matrix. For example, if size-bands were narrower it might be expected that the coefficients on the leading diagonal – the proportion of firms remaining in the same size-band – would be smaller, and (necessarily) some of the off-diagonal coefficients would be larger. However, it is our conjecture that:

1. ‘inertia’ would remain important (more strongly so if growth were being measured by jobs)
2. there would be evidence of a ‘decay’ in coefficients within a size-band which was negatively correlated with size – closer size-band transitions are more likely than further transitions
3. and, comparing matrices between periods, that the leading diagonal coefficients in each size-band would be larger for matrices beyond 5 years than they were up to that age

There are well-known statistical methods for comparing the properties of markov chains (see, again, Lindsey (2004)). So, for example, it is possible to compare the random walk model to a more general alternative (for a common measure of firm growth). Equally, the transition matrix can be allowed to depend on firm characteristics other than size (for examples of applications in demography see Keyfitz and Caswell (2005), and to ‘life histories’ more generally Willekens (2014)).

We have seen that very few of the 239,649 firms born in 1998 (cohort98) survived to 2013, just about 10%. We also know that the bulk of cohort98 firms – 212,427 (90%) – are born with less than five jobs and that these born very small firms make up a similar proportion of the 15 year survivors. Two thirds of the born very small remain very small but amongst the 9,266 which grow, and have 5 jobs or more by age 15, there is an even smaller group, just 1,248 firms, which have grown quite spectacularly: taken together they account for 40% of all jobs added between birth and age 15 by all cohort98 survivors. What if a business support agency had as an objective the early identification of those 1248 firms? Choosing a member of this small group from the cohort98 firms born with less than 5 jobs requires considerable luck: the chance at birth is 1,248 out of 212,427 – about 0.5%.

However we might be able to improve these odds. What if the agency were to select from the firms born very small (with less than five jobs)which have survived to age 5 which *and* ***already have*** *at least 5 jobs*? This narrower selection criterion clearly improves chances dramatically. The the chance of choosing a 20+ jobs 15 year survivor from firms with 5 or more jobs at age 5 is about 9%. This is almost 20 times better than choosing from the cohort at birth, and 5 times better than choosing any age 5 survivor. Moreover, the odds of a firm with 5 jobs at age 5 *dying* before age 15 are also considerably better than those of a smaller firm age 5. Of course, this is not to suggest that policy should be framed this way: selecting firms for support based on a very specific number of jobs at age 5 as a criterion might have unwanted side-effects. 31 Nonetheless, the arithmetic does provide some context for the design of policy and highlights the key role of firm age and size in accounting for firm survival and growth.

This has been, by design, an essentially descriptive study of a very large number of UK firm-level records. Although basing the analysis on the whole population of firms born in a particular year – a birth cohort – and following it over 15 years is not especially innovative, it is certainly unusual and does produce some interesting findings. Of course, it will require the analysis of further cohorts before we can be entirely confident about the robustness of our findings about survival and growth.

## Footnotes

1 Such tables, probably more well-known in sociology, are also known as “mobility tables”. For an introduction see Hout (1983).

2 A 2006 survey paper Santarelli et al. (2006) listed 60 references published between 1962 and 2004.

3 From the concluding section: “... aconsiderable body of knowledge about small firm growth now exists, which is what we tried to highlight in this manuscript. One could easily emphasize the problems instead ....[long list of weaknesses] ... However, the luxury of seeing such deficiencies can only be enjoyed because many researchers put considerable effort into researching firm growth ...” Davidsson et al. (2010, p. 62)

4 see, for example: Kirchhoff (1994); Phillips and Kirchhoff (1989); and most recently Headd and Kirchhoff (2009).

5 A cohort approach is deployed in the Bartelsman et al. (2009, section 1.5.4) cross-country analysis of “post-entry performance”, albeit to a limited extent, and with no mention of growth trajectories.

6 Shepherd and Wiklund conducted a meta-analysis of firm growth studies published between 1992 and 2006 and noted: “Rarely did a study use two or more time spans for calculating growth. ”Shepherd and Wiklund (2009, p. 108)

7 Since some of the businesses ‘exit’(X) there will also be sequences which look like, for example, ‘GDX’, for a business which grows, declines, then exits.

8 Indeed, ‘gambler’s ruin’ is used as a motivating example in many introductory treatments of stochastic processes. For a canonical example see Jones and Smith (2010) where the first introductory probability chapter is followed by a sequence of three chapters (in increasing order of generality): gambler’s ruin; random walks; and markov chains.

9 For an earlier, and (now) slightly dated, summary of evidence for a wide range of countries see Bartelsman et al. (2009, section 1.5.4).

10 Its longevity can, in part, be traced to its policy and/or ‘political’ significance. For a recent retrospective account from one of the participants, see Kirchhoff et al. (2013, pp. 161–163).

11 A conclusion also reached by arecent OECD study, “... it is evident that most micro start-ups either remain stable (i.e., at the end of the period they are in the same size class in which they were at the beginning of the period) or exit the market. In every country, the number of micro start-ups moving to ahigher size class at the end of the period is extremely small – on average around 3% and never more than 8%.” Calvino et al. (2015, pp 27-28)

12 Equally, and by extension, it is presumably this relatively select group to whom ‘models of selection and learning’ apply. For a pair of papers which discuss the composition of the entrepreneurial population see Hurst and Pugsley (2011), and Hurst and Pugsley (2015).

13 The statistical data used here is from the Office of National Statistics (ONS) and is Crown copyright and reproduced with the permission of the controller of HMSO and Queen’s Printer for Scotland.The use of the ONS statistical data in this work does not imply the endorsement of the ONS in relation to the interpretation or analysis of the statistical data. The analysis upon which this report is based uses research datasets which may not exactly reproduce National Statistics aggregates.

14 For a full, official, account of the Business Structure Database and its compilation, see Evans and Welpton (2009).

15 Since an employee can work for more than one firm summing over firms produces an estimate of jobs rather than employment, we ignore this distinction here and use the terms employment and jobs interchangeably.

16 There are some cases where an enterprise comes back to life – its employee numbers fall to zero, and then revive. These records, which are relatively few and are mainly single employee firms, have been excluded.

17 the UK version of the EU NACE rev.1

18 Of course, the fine detail is difficult to see at this resolution, and the latter years are also affected by the ‘great financial contraction’ of 2008/09.

19 The job/firm numbers in each year are all lower than the corresponding job numbers by a constant equal to the natural log of 26 thousand.

20 The contrasting pattern of the early years seems a little anomalous, however it might reflect a difference in the speed with which events are recorded in the underlying business register: the early demise of larger firms may be recorded more quickly.

21 To use an overly simplified biological analogy, the ‘current’ size-band is the ‘nurture’ measure, whilst size-band at birth is the ‘nature’ measure.

22 Of course, some of these firms may have moved up and then moved back, all we can say certainly is that they were not born in any larger size-band.

23 The choice of measurement convention was a key factor in a long-running (still continuing) and acrimonious debate about the contribution of different sized firms to job creation (see for example Haltiwanger et al. (2013)). The US Bureau of Labor Statistics has responded to the issues raised in the debate by devising a compromise between ‘row’ and ’column’ based measures which they refer to as “dynamic-sizing”, see Butani et al. (2006).

24 Though the converse is not universally true: not all firms which drop down a size-band were smaller at birth than those which remain behind.

25 This is where the parallel with the ‘current size’ hazard rate is clear: it is the ‘death’ destination (column) in an origin/destination table which includes all the firms alive in year *t*, not just the firms which survive to (*t* + 1).

26 This conclusion is consistent with a previous study of the distribution of firm growth rates in the UK (all private sector firms, not a single cohort) which found for the two periods analysed (2002/05 and 2005/08) around two thirds of firms recorded growth rates in the -1% to 1% range, see Anyadike-Danes et al. (2009, Fig 7, p. 16).

27 It is worth noting that a parallel analysis of annual proportions computed for *all* live firms – in other words, not just age 15 survivors – yields qualitatively similar findings about the pace of change. In other words, *the relationship between the living is independent of the decline in the proportion dying*.

28 The averages plotted here are averages of the log of the jobs/firm ratios not the log of the average jobs/firm ratios. The averages are therefore geometric means of the jobs/firm data, rather than arithmetic means. A geometric mean is often preferable when there are a small number of extreme values and/or for averaging growth rates.

29 We have in fact converted the slopes of the trajectories (the first difference of the logs) into a conventional percentage change: if *T*_*t*_ is the natural log of a trajectory at time *T* then we compute: [(*e**x**p*(*T*_*t*_ − *T*_*t*− 1_)) − 1] × 100.

30 For a summary account of the evolution of route to this conclusion see Lloyd (2006) and for a discussion of economics more specifically Morgan and Knuuttila (2011). Perhaps more relevant to our work is the position in demography, see Burch (2003) for a trenchantly expressed view.

31 For example it might encourage a four employee firm to hire an otherwise unneeded extra employees in order to become eligible for support.
